# Mechanical Dentistry

**Published:** 1876-11

**Authors:** L. P. Haskell


### MECHANICAL DENTISTRY.

BY L. P. HASKELL.

I have a few suggestions to offer upon the subject of Mechanical Dentistry, that may be of interest and benefit to the younger members of the profession; and as they are the result of thirty years experience in this department, I can speak with some degree of authority.

First, I will call attention to the subject of "bases" for artificial teeth. We should forever discard the practice of using any one of the bases in all cases, for there is no article, be it continuous gum, gold, celluloid, rubber, or the cast metals, that is adapted to every case. This, I am sure, no one of extensive experience will dispute. What is best, in the various cases, is to many a difficult question to decide.

The cast metal plates are desirable mainly for lower plates, where, in some cases, weight is necessary in keeping the plate in position, in consequence of the interference of the muscles, and superabundance of soft integuments. Here I find this material useful as a cheap substitue for continuous gum.

Rubber is useful as a cheap base, enabling many to wear artificial teeth who otherwise would be unable to do so. In many cases of partial sets, however, it is utterly inadmissible. But the great objection to its use is its deleterious effect upon the mucous membrane; and its tendency, from retention of heat under the plate, especially in section plates, to cause undue absorption of the alveolus; so that when the patient can afford a better material, they should be advised not to use this.

Celluloid is a material about which the profession differ in opinion. For myself, from constant use for eighteen months, and the experience of others in whose judgment I have confidence, I may say I still think highly of it, and much prefer it to rubber. My reasons for liking it I will give in brief: Its chief advantage is in its color, enabling the operator to dispense with gum teeth, and by using instead, the plain teeth, secure a more natural arrangement of them, and expression to the features. Then it is stronger than the rubber, and more cleanly to work. It has been objected to on the ground of discoloring in the mouth. I have seen but little of this, and that mainly in the mouths of patients who did not take suitable pains to keep it clean. I do not think celluloid effects the mucous membrane as much as rubber does.

Gold always was and ever will be a desirable base for teeth. For partial sets in a majority of cases, it is the very best material that can be used, making either suction or clasp plates, thin, strong, cleanly, and to which teeth can at any time be added without difficulty. For full sets it is next in value to continuous gum, using celluloid or rubber attachments, especially the former, if plain teeth are used. But this material requires the exercise of skill that is not necessary in the use of rubber or celluloid. Consequently it is a common thing to hear a patient say, "I once wore a gold plate, but now prefer the rubber; it fits so much better." The trouble was not in the material, but in the incompetency of the dentist to use the gold.

There is, however, one exception I will make in favor of rubber or celluloid, over any other material, and that is in the difficult class of cases known as partial lower sets, when success depends upon an accurate fit; in many of these cases it is next to impossible to swedge a plate accurately, but with a plaster impression, and the use of the above materials, an easy fitting, and more generally useful case can be made.

Last, but best, on the list is, "Allen's Continuous Gum," and of course I mean for full sets, as for partial sets I consider it inadmissible. Introduced to the notice of the profession twenty-five years ago. It was then I purchased an office right, and having used it ever since, I can say that in all respects it is the perfection of artificial dentistry, and against it no valid objection can be brought.

But you will say there are objections brought against it. True, and I will answer them. The first objection is on account of weight. Well, it is about time that intelligent and experienced members of the profession discarded the idea that weight is objectionable in artificial teeth. After twenty-five years use of this material, I have failed to find one case where the patient could discover that in the mouth it was heavier than rubber, or that the weight was objectionable, except in some cases where it was not properly adjusted. On the other hand, I have had fewer complaints of its loosening than in the case of the lighter materials.

A prominent physician, who is very critical and exacting, for many years had worn a gold plate, and lately one of rubber, and for whom I recently made a set of continuous gum, said to me after several months experience, "I find the weight of these teeth agreeable rather than otherwise." A lawyer who had worn rubber for several years, and the work was done by one of the best dentists in Chicago, said to me, on having them replaced with continuous gum, "that feels as though it belonged there;" and afterwards said that this set completely overcame the difficulty he previously had experienced in public speaking. This would not have been true had weight been realized. In this connection I would state that the contour of the artificial palate, including the rugas, in a perfect piece of continuous gum, so nearly corresponds with the natural palate, that it assists materially in correct enunciation.

I have had patients complain of rubber, and afterwards wear the continuous gum without the idea of weight occurring to them. What made the difference? Your own judgment will decide.

Another objection often made is, that it "requires too much care and experience." Ah! there's the rub; and it is just there that the profession needs to pause and consider what that objection leads to, viz: that the country is overrun with men calling themselves dentists, who are a curse to the profession, and to the public; your "eight dollar quacks," "tooth carpenters," bunglers and botches.

You, members of the profession, who are striving to elevate the operative department above the reach of these incompetents, tell me who it is that are successful in saving teeth? Is it not those, who having primarily a natural capacity for the business, devote the requisite time to acquire a suitable knowledge of it, and then bringing into exercise all the skill they have, and with careful manipulation, accomplish the best result possible?

It is in this lack of natural ability, proper education, skill, experience, and desire to do the best that can be done, that so many fail. Never offer the excuse for not doing the best that can be done, that "it requires to much care and experience."

Another objection urged is, that it costs too much, and patients are not willing to pay for it. There are in every community, more or less, who are able and willing to pay for the very best of every thing they use; and wherein is the best demanded more than in a set of artificial teeth? Demonstrate to them what they need, and they will have it at any cost.

Some have formed their estimate of this work by specimens from the hands of incompetent men, who have made a failure of it, as they do every thing they undertake. I spare no pains to make each case as strong and perfect as possible, and as a result I have no cases break down; I have far less repairs to make than of any other style of work, in proportion to the number of cases made; and when broken, it is in nine cases out of ten, caused by dropping into wash basins. To avoid this I caution the patient against washing over a basin, but instead to sit down and place a towel in the lap, then if it slips, no harm is done. As to repairing, it is easily repaired and remains as perfect as before breaking.

So much for the "bases." I will offer a few suggestions as to the arrangement of teeth and restoring as far as possible the contour of the face. In these matters many dentists come far short of the requirements of the case. In a large majority of sets of teeth now worn, what do you find? The margin of the plate cut down to a uniform height all around; the incisor teeth and gums very prominent and the canine teeth and fossæ very much depressed. We all know that the canine teeth form a prominent feature of the mouth, making a prominence; the bicuspids retreating from which, and with the molars forming nearly a straight line. After the extraction of the canines, and full absorption has taken place, there is a marked depression which can only be restored by carrying the margin of the plate as high as it can be worn, (and if you have never tested it, you have no idea how high it can be worn, provided you do not leave a sharp edge,) and then building up the fossæ as high as the circumstances require; set the incisors well back, as can only be done in a majority of cases, by using plain teeth, and thus you will be enabled to restore the contour of the face in a great degree.

Lower plates often prove failures from being too wide, so that they are easily displaced by contact with the muscles and the soft integuments; oftentimes the teeth are arranged too far outside the ridge, interfering seriously with mastication.

In articulating the teeth, too much care can not be observed in preventing the interference of the front teeth, and equally of the last molars; the pressure should be upon the bicuspids and first molars. Often, after teeth have been worn a number of years, they become troublesome from no other cause than interference of the front or extreme back teeth. A short time ago, a gentleman called upon me, who was wearing an upper set made several years ago by another dentist in this city. He had been constantly annoyed by the teeth, and thought he must have a new set. A moment's examination revealed what I had imagined might be the trouble; a constant interference in front, throwing the plate off whenever the mouth closed. I remedied by grinding, and now his teeth give him no further trouble; and yet his dentist, after repeated interviews, failed to comprehend the difficulty.

Patients are often annoyed by things that can easily be remedied, if the dentist will only look in the right direction; as for instance, a plate hurts, and he cuts and files the plate, when the trouble is owing to undue pressure from the teeth being to long on that side, and what is needed is a little grinding of the teeth, and not filing of the plate.

